# Interspecies Relational Theory: A Framework for Compassionate Interspecies Interactions

**DOI:** 10.3390/vetsci12060586

**Published:** 2025-06-14

**Authors:** Emily Kieson

**Affiliations:** Equine International, Boston, MA 02115, USA; ekieson@equineintl.org

**Keywords:** animal–human bond, intraspecies relationships, animal–human communication, animal-assisted, animal welfare, wellbeing

## Abstract

Most studies on relationships between humans and nonhuman animals focus on the benefits of the relationship to humans, the potential detriment or stress to animals, or how humans can better improve husbandry or handling practices in the domestic setting. By comparing existing research in human working relationships and friendships with animal friendships and studies on human–animal interactions, this paper proposes a new framework of an Interspecies Relational Theory that provides approaches for identifying, developing, and maintaining different levels of relationships between humans and nonhuman species. The framework is broken into three stages aligned with existing research in human levels of trust ranging from strategic/calculus-based to working/knowledge/information-based to friendship/affect-based. These levels are also aligned with stages of Maslow’s Hierarchy. The suggested framework can help provide insight into both human and animal perspectives of the human–animal relationship in a variety of veterinary and animal-assisted settings.

## Simple Summary

There is a rising interest in the welfare and perspectives of animals in domestic settings and within animal–human interactions. Most studies involving humans and animals focus on either the benefit to humans or the impact on animals. By combining studies in human friendship with knowledge on animal friendship, communication, and animal–human interaction, this paper introduces a new framework around interspecies relationships, how they are formed, and how they are maintained. The process is broken down into stages of the relationship corresponding to existing models of human relationships and behaviors and perspectives on trust and social bonding, providing a basic framework for approaching and building relationships between humans and nonhuman animals.

## 1. Introduction

Most studies on relationships between human and nonhuman animals focus on the benefits of the relationship to humans, the potential detriment or stress to the animal, or how we, as humans, can better accommodate husbandry welfare protocols for domestic or captive animals [1,2]. Even in the context of animal-assisted services, most of the literature focuses on the impact of interactions and animal–human relationships on humans [3], with little discussion around the experience for the nonhuman animal. More scientists, however, are shifting the focus of research and its application to understanding components of relationships between humans, nonhuman animals, and environments from all perspectives [2,4,5] to help improve our understanding of both human and nonhuman animal perspectives in all areas in which human–nonhuman animal interactions occur. For the purpose of this paper, the author will use the term nonhuman animal to emphasize the importance of mutuality within the relational context. The term “animal”, however, will be used if the cited reference uses this word instead of nonhuman animal.

Even in animal-assisted work where relational dynamics are often seen as the justification for incorporating animals into therapeutic practices, most physical interactions between human and nonhuman animals are more in line with instrumentalizing the animal (as a form of exploitation) rather than as a partnership [6,7,8,9,10,11]. Since the animal–human bond is considered a core foundation of these services, the client’s subjective experiences of the relationship with the nonhuman animal are considered essential for the outcomes of animal-assisted services. The interactions between clients and nonhuman animals, however, do not always align with these relational goals. In 2018, Chandler [3] introduced the Human–Animal Relational Theory (HART) to help practitioners in animal-assisted mental health better understand the relationship between humans and animals from the human perspective to better facilitate therapeutic outcomes. The nuances of the relationship and how they develop for the nonhuman animal, however, are not discussed within the HART paper. The literature on animal-assisted services often cites the relationship between human and nonhuman animals as a primary source of therapeutic outcomes [3,12] but little work has been performed to consider how this relationship develops in ways that align with theories in relational development or friendship from both the human and nonhuman animal perspectives. Although researchers have studied friendships in nonhuman animals, research in intraspecies friendships in nonhuman animals is relatively new. Existing measures of friendships in nonhuman animals include both behavioral and hormonal indicators, with results suggesting that proximity, time, and species-specific behavioral interactions are key indicators of friendships in nonhuman animals [13,14,15,16].

Recent research acknowledges that nonhuman animals live in complex environments and many studies in animal behavior have changed how we view and understand culture in nonhuman animals. Current animal social behavior studies suggest that nonhuman animal social species generally live in cooperative settings, avoid conflict, and engage in reconciliation to maintain order, reduce overall long-term stress, and maintain social relationships [17,18,19]. Research on social networks and pro-social behaviors in nonhuman animals often builds on prior studies of cooperative interactions and reciprocity [20], where behavioral indicators of affiliative bonds, such as those resembling friendship, parallel findings in human social relationships, offering potential insights into the structure of nonhuman animal societies. Trust is a necessary component of these social structures and plays a fundamental role in how we integrate animals into our lives. Although companionship is an important part of the human–animal bond, in Western culture we also build trust in human–nonhuman animal interactions with the deliberate intention of breaking it to serve human needs [21]. Interactions and management styles, especially under laboratory, agriculture, and even some domestic settings focus almost exclusively on the needs of humans rather than interspecies mutuality or relational growth. This includes husbandry, handling, and training where the desired outcomes are focused on serving human needs rather than the relational growth between humans and nonhuman animals.

Interest in the relational side of the animal–human bond from the nonhuman animal perspective has grown and its importance in domestic and captive settings continues to develop. Research on animal welfare has improved in recent years, including the expansion of the understanding of the five domains to include mental state and animal–human interactions [22]. Concepts of one welfare have also risen in popularity with more thought given to how human actions impact the environment and nonhuman animals [4] and how these impact our relationships within our homes [23,24]. The complexity of the interactions and impacts on the nonhuman animal are not always considered, however [2], so it is critical to understand the elements that contribute to the relationships that develop between humans and nonhuman species. A greater understanding of the social relational dynamics between species allows us to better recognize the ways these contribute to the wellbeing of humans and nonhuman species in domestic and captive environments. This is especially important in veterinary and animal-assisted settings where interactions are almost guaranteed. However, despite the widespread use of human–nonhuman animal interactions in therapeutic and domestic contexts, few frameworks exist that conceptualize these relationships as co-developed through mutual experiences and stages of trust. This paper addresses that gap by offering an integrative model grounded in cross-species behavior, trust theory, and welfare considerations.

This paper is a theoretical review that integrates findings across disciplines to propose a novel framework for understanding how trust and relational development occur in interspecies relationships, proposing Interspecies Relational Theory as a framework for understanding compassionate and equitable interactions across species. The theory organizes key components of relationship development between humans and nonhuman animals, particularly within domestic contexts. While the primary focus is on human–nonhuman animal relationships, the framework is intended to be adaptable to other interspecies contexts where mutual social bonding and communication are possible.

The following sections present the theoretical alignment with Maslow’s Hierarchy of Needs, explore the stages of relationship development, and examine implications for welfare, training, animal-assisted services, and veterinary practices.

## 2. Alignment with Maslow’s Hierarchy

The order of building interspecies relationships aligns with ideas set forth in Maslow’s Hierarchy of Needs as it applies to all animals. In the Hierarchy, physical needs must be met first, as no higher-level development can occurunless physical needs are met. This includes air, water, and food [25]. If a nonhuman animal (or human) has insecurities around basic needs, it may be difficult to consider forming or maintaining more complex social relationships. If basic needs are met, however, then additional physical and psychological factors can be considered and used to build the foundation of relationships, both within and between species.

The second tier of the Hierarchy focuses on safety. In the paper by Griffin et al. [25], dogs’ safety needs are described as having physical health and safety, having choices/having agency, feeling physically safe, and feeling psychologically safe (predictable environment). The five domains [22] also include these options when discussing welfare in animals as primary components of animal welfare, especially when looking at welfare from the perspective of the nonhuman animal (domain 5: mental state). While humans can provide what is considered a safe and healthy physical environment, it is ultimately up to the nonhuman animal to decide whether they feel safe in that environment. The subjective sense of both physical and psychological safety therefore serves as the foundation for concepts of trust within Interspecies Relational Theory. As a larger concept, however, trust is broken down into stages that build over time and overlap with other elements of relationships that cover the higher levels of the Hierarchy. At the lower levels of Maslow’s Hierarchy, the concepts outlined in Interspecies Relational Theory cover the development of subjective feelings of physical safety, building a sense of psychological safety through consistent predictable interactions including the development of mutual language/communication, and consistent exchanges of interactions over time that align with expectations and repercussions.

The third tier of the Hierarchy encompasses love and belonging or, more specifically, social needs [25]. In this category humans and nonhuman animals (especially those belonging to social species) seek out social interactions and companionship. In Maslow’s Hierarchy of needs for humans, this category includes love and belonging in a way that goes beyond just companionship and includes levels of emotional bonding. When overlapping Interspecies Relational Theory with this level, we move beyond basic physical trust and exchanges and into emotional support in ways that align with ideas of the human–animal bond. It is also at this level where humans and nonhuman animals seek emotional safety with others, especially when environmental or social conditions threaten the subjective sense of safety (the lower tier of the Hierarchy).

While Maslow’s Hierarchy offers a valuable model, recent critiques emphasize that needs may not always follow a strict hierarchy, especially across species with different environmental and evolutionary pressures. Although recent critiques have suggested alterations to Maslow’s Hierarchy, they still suggest that individuals will often seek out fundamental needs such as water and physical safety prior to social connections [26]. Revisions of the Hierarchy also emphasize the overlap of levels and the constant assessment of many levels at once rather than a linear hierarchy and the importance of recognizing subjective experiences that can shift the importance of the levels [26,27]. Even with criticisms, the Interspecies Relational Theory aligns with most models of the Hierarchy concept, and so the following structure of relationships will refer to Maslow’s Hierarchy with the understanding that there is fluctuation and subjective changes in motivation that may shift this perspective for each individual.

## 3. Relationship Stages

All stages of relationships are defined by different levels of trust and interactions. Trust, as defined by Schilke et al. [28] can be described as “the willingness of an entity (i.e., the trustor) to become vulnerable to another entity (i.e., the trustee). In taking this risk, the trustor presumes the trustee will act in a way that is conducive to the trustor’s welfare despite the trustee’s actions being outside the trustor’s control.” In a broader sense, trust can be defined as a willingness to be vulnerable to another party based on the expectation of positive behavior or intentions [29].

This concept of trust builds on the relationship lens using Maslow’s Hierarchy; if the feeling of safety is not present, then concepts around trust cannot be established. Trust in physical safety therefore needs to be established prior to other levels of trust.

When considering trust within a relational setting, social sciences focus most often on the role of trust in human–human interactions and how humans may carry this perspective into human–nonhuman animal interactions. In these contexts, the concept of trust usually starts with concepts of “trustworthiness” and abstract and subjective ideas of trust prior to interactions [30]. When defining trust from both human and nonhuman animal perspectives the concept should be broken down even further. Rault et al. [31] provides insights into how humans can gain insight into the nonhuman animal’s perception of relationship quality by observing behavioral signals and indicators of positive experiences based on interactions. These signals will ultimately differ based on species and individualized expressions.

### 3.1. Stage 1: Physical Trust, Communication Development, and Initial Engagement

#### 3.1.1. Trust—Physical Safety

**Figure 1:**
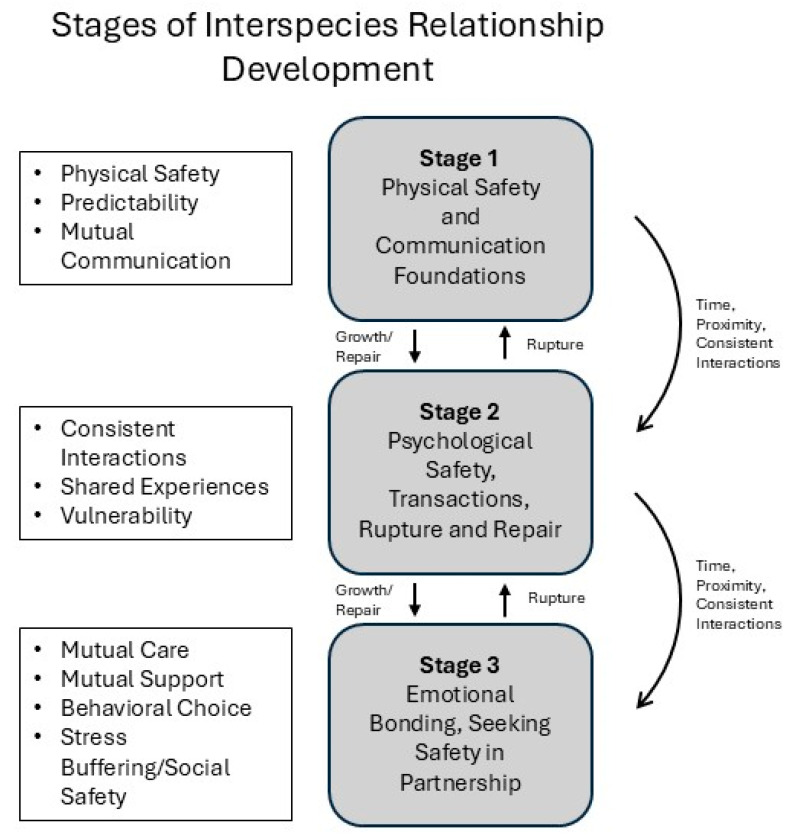
Visualization of each stage of relational development within the Interspecies Relational Theory. Lists provide summaries of each stage of relational development and arrows indicate direction of development based on growth or rupture and repair.

All animals, humans included, must assess physical threat prior to engaging in trust at higher levels. In trust research in humans this level can be called strategic trust or calculus-based trust [32,33], which is where basic physical safety is established and individuals recognize that the other party is not a threat (Figure 1). This stage also includes a level of fragility where trust is subject to changes based on continued interactions, exchanges, and experiences [34] and basic concepts of trust within interactions that align with threat assessment (physical safety). For nonhuman animals, this stage is established when either the fear response is evident via avoidant or flight behaviors (or individualized expressions including freeze or fawn/please), or the animal is willingly to engage of their own volition. Generally, an individual who does not feel safe and has not yet established their level of trust will either cease to move towards or move away [35]. The engagement and subjective establishment of physical safety is evident through behaviors indicating curiosity and exploration that help individuals of all species to acquire new knowledge about novel objects or beings [35].

#### 3.1.2. Associated Interactions with Physical Safety

Each individual within the interaction must be allowed to develop their own sense of physical safety and be allowed to express behaviors that provide insights into their subjective experience. Since physical proximity aligns with physical safety, it is important to allow freedom of movement to provide individuals with the ability to choose the level of space needed to feel physically safe. Knowing the behavioral expressions of fear, including species-specific and individual expressions of fight, flight, or freeze (or appeasing) responses is also helpful at this stage to assess the subjective experience of the other. This also means assessing behavioral responses at different proximities and adjusting space to allow for increased feeling of safety. Ideally, enough time and space are given to allow for the development of subjective feelings of physical safety, at which time additional elements, such as novel objects or food, can be added to encourage seeking behaviors. For veterinary practices it may take some time to develop this level of trust between a worker and a nonhuman animal client. In this case, utilizing the animal–human bond developed between the owner/caregiver and nonhuman animal can provide the needed sense of social and emotional safety for the nonhuman animal client.

#### 3.1.3. Associated Interactions with Building Mutual Language and Communication

Language and communication are built through the consistency of interactions with predictable outcomes [36,37]. At this stage only preliminary language and communication are developed, potentially as means of establishing basic understanding of threat assessment, safety, and intention. The pace at which language is built between two individuals is unique to the pair and may develop fast or slow depending on past experiences. With regard to horses and humans, for example, horses can read facial expressions, vocal cues, posture, movement, attention, and even odor from humans [38]. By recognizing signals we intentionally set and ones that are less intentional, we can start recognizing the various forms of communication that occur between us and nonhuman animals that may impact relationship development. Through the establishment of consistent interactions and corresponding consistent responses, each individual can better assess the intentions of the other based on approach, retreat, and other behaviors that indicate intention, motivation, or emotion (including fear). Examples of how this plays out between humans and equines can be seen in the case study written by Mealand et al. [39] where proximity, time, communication, and safety serve as fundamental pillars of building interactions with horses in the holistic equine learning plan (HELP) model.

#### 3.1.4. Additional Considerations for Physical Safety and Building Mutual Language and Communication

At this level, individuals assess physical safety through time and physical proximity and continue development of consistency through shared communication. Proximity and physical safety are still assessed, and the closer the individual, the higher the potential threat. How an individual approaches and maintains proximity can provide additional information as to the subjective experience of safety of the individual, with fear responses often resulting in behaviors that decrease proximity. This is often referred to as the “flight zone” [40]. When possible, it is important at this stage to be educated on the species-specific behaviors of nonhuman animals within the interactions to improve the progression of communication building and safety assessment of the nonhuman animal.

Communication and shared language are being developed at this stage, so it is critical to be as consistent as possible when building a shared language. Predictability is also constantly assessed since the relationship is still based on limited interactions. In this case, safety is closely tied to predictability meaning that inconsistent communication reduces predictability and may consequently be perceived as less safe. If physical interactions occur at this stage (or even movement and proximity-based communication), great care should be taken to maintain consistency to achieve mutual understanding. For those in veterinary or zoo environments, this means any interactions, especially those involving positive reinforcement or other operant conditioning tools, should be carefully implemented.

It is especially important to be informed on behaviors indicating species-specific behaviors related to seeking (curiosity and exploration) or fear. Behaviors, however, can vary based on individuals and individual history so, where possible, observing the individual in their regular environments prior to engaging in interspecies relationships can provide essential insights into how the individual may communicate and respond in new relationships. The development of physical safety can take time and the length of time an individual feels safe with another can be subjective and dependent on the individual’s history and past experience [41]. At this stage, since trust has not yet been established, if the subjective sense of physical safety is broken it may take longer to reestablish due to lack of previous positive experiences shared between the two individuals. It is therefore important to take care at this stage and build this stage at the pace at which both individuals (human or nonhuman animal) feel comfortable. These are the earliest interactions and form the first associations within associative learning. These are the only interaction upon which each individual can base the understanding of the relationship and basic assessment of safety. Repeated safe interactions build to create patterns of associative learning around a sense of physical safety or, at the very least, lack of threat.

For practical application it is also important to note that there is no definitive timeline for this stage, although calculated reward training and gentle handling, such as those used in cooperative care [42] and “fear-free training” [43,44], can be used to conduct necessary interventions at this stage. Positive interactions that increase the possibility for the free expression of curiosity and seeking behaviors allow for the ongoing sharing of space, building of communication, and time and proximity for shared interactions that build towards Stage 2. The willingness to seek closer proximity is an essential part of shifting from Stage 1 to Stage 2.

### 3.2. Stage 2: Psychological Safety, Ongoing Exchanges, and Rupture and Reconciliation

#### 3.2.1. Trust—Psychological Safety (Subjective Sense of Safety)

Once physical safety has been established, individuals build trust through exchanges and repeated transactions where predictability, reliability and, in the case of humans, empathy is demonstrated [45]. After individuals have developed basic communication in Stage 1, communication is further developed at this next stage. Communication in Stage 1 focuses primarily on expressing subjective feelings of physical safety and exists mostly to convey information regarding seeking (curiosity and exploration) or fear (moving away). These seeking and fear behaviors would be directly related to the other individual in the interactions, with seeking often being expressed as a behavior moving towards the other and fear being expressed as either moving away from the other, freezing, or in a defensive (or aggressive) response (although a please response may also occur). At this next stage, if physical safety has been established through repeated interactions (aligning with associative learning), additional communication may be developed that aligns with other emotions such as play, lust, grief, or even caregiving. These other areas of emotions suggest a social–emotional engagement with the other individual within the relationship [46]. Communication is therefore further developed at this stage by each individual continuing to express new motivations and emotions through the ongoing development of a shared language through shared interactions consisting of consistent exchanges and responses (Figure 1).

It is also at this stage that trust can be broken through inconsistent interactions or exchanges. When something happens to undermine the sense of safety, whether physical or psychological, the line of consistent positive and safe associative interactions becomes broken and one individual will choose to no longer participate in current or future interactions. The ability to assess and choose continued interaction is dependent on the animal or human having agency within the interaction and communicating to the other that they no longer choose to participate (through shared communication strategies around safety preliminarily developed in Stage 1). An example of interspecies interactions of trust at this level can be seen in the paper by Pelgrim et al. [47] where domestic dogs were subjected to trustworthy vs. untrustworthy humans during communication interactions. After experiences with both types of humans, the dogs, when given choice, chose not to respond to the untrustworthy human.

The role of autonomy and choice provides each participant with the ability to express behaviors indicative of their subjective perspectives of the interactions and the stage of the relationship. As behavioral exchanges across relational stages foster mutual understanding, the preservation of agency and choice is essential to ensure that both participants remain active co-creators of the relationship.

#### 3.2.2. Associated Interactions in Creating Exchanges, Rupture, and Reconciliation

Once communication and language have been established, individuals can start engaging in more complex interactions and exchanges. This level parallels with working trust as defined by Lewicki and Bunker [32] and further supported by Mayer [29] and knowledge-based trust as explained by McAllister [33]. Interpersonal interactions between people also show that trust builds over time based on repeated sequences of interactions that are dependent upon learned associations based on those repetitions and multiple levels of understanding (cognitive, emotional, social, contextual, etc.). The ongoing development of communication can lead to a greater understanding of each other’s goals and the building of mutual goals (information-based trust) [33]. This includes when one or both individuals experience uncertainty (whether in or outside of the relationship) and trust is either tested (as a means of support when one feels uncertain) [48] or rebuilt after conflict (rupture and repair) [49] (Figure 1).

In nonhuman animals, this stage includes the building of exchanges and associations learned through repeated interactions between nonhuman animals or between humans and nonhuman animals. For humans this process may include positive feelings around nonhuman animals at home as well as parts of animal-assisted services [50,51,52], although there is no research that the feelings are reciprocated by the nonhuman animal in these conditions which suggests that the relationship between human and nonhuman animals may not be mutual.

In livestock this often includes positive interactions or the willingness of humans to slowly habituate animals to new stimuli. In cattle, for example, students who took the time to work with cows to habituate them to novel objects combined with positive human interactions had more favorable outcomes when faced with new challenges [53]. Horses in unfamiliar or stressful situations showed lower stress responses when a familiar handler was nearby [54], suggesting a sense of safety through familiarity but not enough to suggest a social bond.

After repeated positive associations have been built, new levels of interactions can occur within this level of trust. More complex communication allows for more complicated language patterns. This can provide additional insights into the motivation and emotions of the other but also increases the likelihood of miscommunication and a disruption of trust. At this level the subjective sense of psychological trust may also fluctuate, partially as a result of this ongoing building of language, but also due to the ongoing growth and uncertainty of how the relationship fits into the wider social world of the individual. The ongoing associative learning of acting and responding aligns with economic exchanges and transactional interactions. This is where the relationship may shift from information-based to knowledge-based [33] to facilitate ongoing collaborations that are mutually beneficial. Within the research of human–nonhuman animal interactions, examples can be seen where fishermen and dolphins have learned to work together to increase catch and feeding opportunities on local fish [55]. Wild greater honeyguides have also partnered with humans to collaborate in finding beehives where both benefited from the harvest [56]. These interactions are also in line with concepts of operant conditioning, which is where one individual deliberately provides consequences, whether reinforcement or punishment, for behaviors that are either desirable or undesirable in the other [57,58]. Operant conditioning and behavioral shaping are forms of transactional interactions where interactions are initiated with an expected outcome from the other. This transactional interaction, when using operant conditioning to either add or remove rewarding or aversive stimuli, includes levels of physiological arousal [59,60]. The introduction of such stressors also increases the likelihood of rupture and repair.

At this level of development, uncertainties and ruptures are inevitable due to the inherent unpredictability of environments and responses/reactions. Stress, whether intentionally integrated into the interactions or accidental, will be an inevitable part of a relationship between two beings [61]. This includes conflict, social disruptions, or disagreements. According to research in human psychology, the role of trust becomes even more important as the degree of conflict or relational disruption increases [62], suggesting that, in order for a relationship to withstand a level of stress, the relationship will have first needed to have built an equivalent level of trust.

The resolution of these stressors will come into play at this stage of trust and relationship building. Conflict resolution, or the temporary dissolution and restoration of trust, is evident in most social species, with preferred partners often engaging in affiliative behaviors after conflict [17], a behavior aligned with social stress responses of tend and befriend [63,64,65,66,67]. At this point, communication may default back to the communication developed at Stage 1 to both express and assess levels of physical stress.

When human relationships are disrupted due to conflicts, individuals are likely to attempt reconciliation if the future of the relationship requires ongoing connection and collaboration [68]. Reconciliation (affiliative behaviors that occur between two members who were in conflict) also takes place between other social species such as lemurs [69], other nonhuman primates [70], wolves [18], and other social species [17].

The disruption of trust can occur as an accidental occurrence (e.g., environmental stimuli that becomes associated with the partner) or because of interactions within the dyad. The disruption of trust may be accidental (e.g., accidentally tripping on a cat) or deliberate (the use of positive punishment or painful aversive negative reinforcement in training). The other in the pair may not know if the occurrence of aversive interactions was deliberate or accidental potentially leading to a disruption of trust. For example, dogs are less likely to follow the cues of an inconsistent informant when following signals for treats [47,71], suggesting that previous interactions build on trust. Furthermore, dogs from aversive training programs showed more stress behaviors than those from positive training methods [72] potentially linking stress with human interactions and affecting trust towards humans. Additionally, as a means of rebuilding and reconciling after conflict, dogs have been shown to demonstrate affiliative behaviors towards humans after conflict as a sign of reconciliation [73].

Trust building, disruption, and reconciliation are not limited to humans and domestic nonhuman animals. Some species of cleaner fish have evolved to learn how to cooperate, demonstrating that fish can develop levels of relationships with different species [74]. Cleaner fish are more likely to engage in beneficial (less cheating) behavior when interactions are more regular with clients [75] aligning with predictability and consistency in building trust and working relationships and transactional interactions at this stage. Additional experiments with cleaner fish also indicated that feeder fish can learn from the behavioral responses of their clients under experimental conditions if their behavior is undesirable [76], thereby building trust or reconciling.

#### 3.2.3. Additional Considerations for Psychological Safety, Exchanges and Transactions, and Rupture and Repair

At this level physical safety is still assessed over repeated exposure (time) spent in closer proximity to the other. This stage may include more physical interactions where touch (from humans or others) is often part of the interaction. Closer proximity creates opportunities for physical contact and, since physical safety has been established, this closer proximity lends itself to more nuanced interactions. For humans, this would include where humans would physically engage with nonhuman animals during animal-assisted services, where animals would request to play with another, or where veterinary nurses would physically engage with the nonhuman animal client.

This stage also includes a willingness to be more physically and psychologically vulnerable as the sense of safety increases. In this sense, the ability for the animal to choose the type and duration of interaction may also play a larger role in the continued development of trust based on ongoing mutual communication [77]. It is important at this stage to not only be informed of species-specific and individual expressions of seeking and fear, but also of play, grief, caregiving, and lust. Additional knowledge in individual behaviors can still be acquired through observations but will also emerge as part of mutual building of shared communication strategies.

Much like in Stage 1, the level of disruption the relationship can withstand is only as great at the level of trust achieved (i.e., the relationship cannot withstand more disruption than the level of trust built to this point). Many relationships may stay at Stage 2. Interactions at this stage are mutually beneficial, even if they are largely transactional. Relationships evolve to Stage 3 only if both parties willingly choose to involve the other in deeper levels of trust. It is possible, however, that one individual may feel the relationship is at one Stage while the other has a different understanding. This is often the case with human–nonhuman animal relationships where humans may feel more bonded to the nonhuman animals than the nonhuman animal does to them. Animal-assisted services are often focused on the human–animal bond based on human-centric outcomes and, in the case of horse-human interactions, humans often feel emotionally bonded with their horses despite behavioral interactions remaining as transactional and often human-centered [78,79].

### 3.3. Stage 3: Deeper Relationship and Friendship

At the deepest level, two individuals establish a relationship that is based on mutual vulnerability, emotional affect, and shared support [33,80]. Repeated interactions over longer time periods provide not only associations and predictability (or lack thereof), but also facilitate opportunities for alignment of values, mutual goals, and emotional affects (affect-based trust) [33] that can result in both emotional and physiological changes in partners [81,82]. When considering the quality of the components of friendships at this level, human research indicates that support of the other’s goals and autonomy are among the most important components of friendship [83].

At this level, two bonded individuals have built physical safety and have undergone the slow growth of psychological safety. This includes the experience of rupture and repair that helps individuals recognize a pattern of predictability of physical and psychological safety within the relationship despite uncertainties that occur within or outside of the relationship. Under these circumstances, humans and nonhuman animals will associate their friends with physical and psychological safety if other uncertainties are present. This means they will gravitate towards their friends under stressful conditions to find comfort in social interactions [63,64,65,66,67].

#### 3.3.1. Associated Interactions Focused on Time, Proximity, and Seeking Safety

During the initial stages of relationship building, trust was built though the repeated choice of spatial sharing (proximity) over time, thereby creating associations of close spatial proximity with both physical and psychological safety. At this stage, time spent in close proximity is also an indicator of close partnership and strong social bonds. This is true for intraspecies relationships in humans and nonhuman animals [84,85] as well as in human–nonhuman animal pairs [86,87]. The desire of one to seek closer proximity and remain in close proximity to another has also been observed between cats and humans [87], dogs and wolves and humans [86], and horses and humans [54].

At this stage one individual will seek out proximity or interactions with the other as a means of seeking safety and comfort. In Stage 1 communication was developed around physical safety in relation to one another. At that stage, individual experiences of fear and the corresponding behaviors were evaluated in response to the other individual within the relationship. In Stage 1, fear responses would result in one individual moving away (increasing distance) from the other. At Stage 3, the stability of the relationship and the other individual within the relationship are solidified as safe and comfortable. When an individual experiences fear in response to environmental stressors or external threats, the associated emotional response will result in behaviors that bring the individual closer to their relationship partner rather than farther away. The tendency to seek close proximity and engaging in affiliative behaviors with friends during or after stress reflects the tend and befriend response, in which individuals display social behaviors more frequently or with greater intensity toward preferred partners during and after stressful conditions [65]. This social behavioral response has been seen in both human and nonhuman animals [63,67], suggesting that it is likely to play a role in human–nonhuman animal relationships that develop to this stage.

#### 3.3.2. Additional Considerations for Developing Deeper Relationships

Although the Interspecies Relational Theory applies to any two individuals, human or nonhuman, who engage in social bonding, the application of these principles is especially important when considering the human–animal bond. The human–animal bond, often characterized as a mutually beneficial and dynamic relationship [88], can be particularly evident at this level of friendship, where shared experiences and emotional attunement (created through mutual language and repeated positive safe associations) form the basis of long-term affiliative partnerships. Research in this bond, often studied through subjective experiences in humans and behavioral and physiological measures in nonhuman animals, focuses almost exclusively on how this relationship impacts and is perceived by the human [12,89,90,91], with limited evidence suggesting that nonhuman animals experience the same level of emotional connection at this stage. Most research on the nonhuman animal experiences of social emotional bonding within the animal–human bond focus on dog–human relationships. There is evidence within these studies to support that dogs experience emotional connections with specific humans (often with whom they have a rich shared history) [92,93]. This suggests that deep, mutual emotional bonds can occur between nonhuman animals and humans, however further research is needed to understand the conditions under which these bonds develop and to identify specific indicators for assessing the nonhuman animal’s perspective. Additional perspectives on the human–animal bond refer to the influence of biophilia and social support as contributing factors, especially for the human [94]. By proposing a theory that encompasses all species and interspecies relationships, we can develop perspectives and models that center equity between participants. Interspecies Relational Theory offers a framework in which both human and nonhuman animals are recognized as equal agents within the relationship, with outcomes and experiences defined by each individual according to species-specific and individual behavioral and physiological parameters.

## 4. Additional Factors

### 4.1. Subjective Experiences

Relationships and the building of relationships is a subjective experience since the stages often play out differently based on species-specific behaviors and individual differences. The subjective experience plays a key role in domain 5: mental state of the five domains [22], which includes how nonhuman animals experience interactions with other nonhuman animals as well as with humans. Disruptions to social development as well as past experiences with developing safety and relationships can also impact how an individual responds to relationship building at each stage. For example, the types of shared history between horses and humans influences the individual relationships, with both positive and negative experiences affecting future interactions [38,77,95,96]. With regard to horses, Merkies et al. [38] emphasize the importance of umwelt in horse–human interactions and the role of not just individual experiences, but also the senses and perceptions of horses in horse–human interactions. Past experiences, both positive and negative, can impact how individuals continue to build interactions and associations within the relationship as well as how these may generalize to how relationships are built with others. Acknowledging behavioral patterns within and between relationships can help provide insight into not only how individuals have built and responded to relationships in the past, but also how they are likely to behave with others in the future.

### 4.2. Hormonal Correlations

Research in neurobiology and endocrinology provides insights into some of the hormonal markers of emotional bonding. Research has shown that both oxytocin and vasopressin play important roles in familial bonding and friendship [97,98,99,100,101] while cortisol can often synchronize between two members in a bonded pair [102]

The same hormones play important roles for nonhuman animals too. Much of the research in humans was originally performed on animal subjects and so there is already a large body of research on the roles of oxytocin and vasopressin in rodent species used in psychological research, especially prairie voles [103]. Research in rhesus macaques suggests that oxytocin and vasopressin are also present and provide hormonal indications of social bonding with friends [104]. Additional research suggests that oxytocin promotes social proximity (and decreases conflict) in lions [105].

In domestic species, recent research has focused on the role of oxytocin in dogs and humans in social contexts. Dogs, unlike wolves, engage in eye-contact that can increase oxytocin and affiliative behaviors between dogs and their owners [106]. As part of the co-evolution of dogs and humans, this cohabitation and relationship development between humans and dogs may have contributed to the findings that dogs are less likely than wolves to seek reconciliation with another dog after conflict [18] but will seek reconciliation with humans [73]. In a study of dogs with owners, the co-evolution of some dog breeds combined with long-term relationships with a human resulted in cortisol levels that related to both the relationship with the owner as well as the owner’s personality [107]. A well-known study by Nagasawa et al. [106] showed a positive loop of oxytocin when owners and their dogs shared eye contact as a means of shared social engagement. With regard to interspecies relationships it is important to recognize the role of hormones and their effect on behavior and emotional associations.

### 4.3. Framing Training and Veterinary Treatment in Interspecies Relational Theory

Animals can experience levels of stress (whether excitement or distress) during structured human interactions involved in structured training (operant conditioning) or regular handling practices such as those in veterinary clinics. It is important, therefore, to prepare nonhuman animal partners for these varied types of interactions. Training, handling, and even some animal-assisted services inherently include close proximity, levels of uncertainty, various forms of communication, and often physical touch. With this in mind, handlers and practitioners should take great care to assess where participants (human and nonhuman) feel with regard to Stage 1. Any nonhuman animal expected to partake in these encounters should therefore be allowed to establish levels of trust equal to those expected of them during the interaction. The required level of interaction should therefore match the level of training and take place prior to exposing the nonhuman animal to these potential stressors. This also includes providing adequate choice and room to adequately adapt to the “discomfort” and stress that are part of training and procedures. This means building the levels of trust and relationship that can withstand these levels of stress. It also means building towards a friendship where nonhuman animals can find safety and comfort in their human companions during stressful or uncertain occasions. If we, as humans, are the ones acting in strange and potentially unpredictable ways, it is important to recognize the level of relationship that has been built and then engaging in reconciliation (from us to them) to build back trust in the appropriate ways and at the appropriate speed tailored to the individual.

Sometimes, often during veterinary practices, there is not enough time to allow for nonhuman animals to engage in interactions with full autonomy. In these instances, relational interactions often involve levels of stressors (usually types of handling or veterinary procedures) that might otherwise exceed the level of trust built between the human and nonhuman animal. In these specific circumstances, the nonhuman animal is expected to comply with interactions and practitioners often expect or desire specific behaviors that help ensure the safety of all involved. Ensuring high levels of consistency in interactions within Stage 2, especially those aligned with positive reinforcement within operant conditioning, can provide levels of predictability and consistency that continue to build the relationship through transactional interactions. Evaluating appropriate reward values, reinforcement intervals, and timing are key to preventing frustration and distress [108].

These types of interspecies stressors and reconciliations are not unique to the relationships between humans and nonhuman animals. The cleaner fish experiment by Bshary and Grutter [76], for example, provides some insights into how animals can use behavioral shaping with each other when they find the behaviors of their partner to be misaligned with their own desires. As with both humans and nonhuman relationships, a foundation of trust must be established prior to adding stressors, and reconciliation is necessary if that trust is compromised. Given the growing emphasis on low-stress handling and welfare in veterinary medicine, this framework offers a structured lens for understanding how relational development can mitigate stress responses, enhance compliance, and support emotional resilience in clinical settings.

### 4.4. Animal-Assisted Services and the Animal–Human Bond

In contrast to veterinary work, when considering animal-assisted services the development of the relationship should come before any expected behavioral compliance of the nonhuman animal. The animal–human bond often serves as the focus of how animal-assisted services provides benefits to humans and a greater understanding of the interspecies relationship can improve insights into interactions that align with intra- and interspecies relational dynamics. How individuals form relationships with other animals (human or nonhuman) provides insight into how they have formed relationships in the past and how they might carry these behaviors into future interactions. It is therefore essential, for both humans and nonhuman animals, to understand the dynamics of how these relationships are formed, the subjective experiences of both human and nonhuman participants, and what types of relational interactions are being supported or encouraged during these interactions.

As with any relationship, stressors are an inevitable part of building interspecies relationships within animal-assisted services. However, these moments should be allowed to progress naturally, with both the human and nonhuman animal maintaining the freedom to make choices about the relationship’s progression. This approach supports deeperpractitioner understanding of the relationship and fosters learning opportunities in relational development. Both human and nonhuman animals need to have high levels of agency and choice within this dynamic, however, to provide full insight into how both individuals choose to develop their interspecies interactions. It is also critical to understand both human and nonhuman behaviors that align with emotions and relational skills to support healthy interactions, intervene to ensure good wellbeing for all individuals, and create opportunities for reflection.

Human–nonhuman animal interactions and the animal–human bond serve as the foundations around which animal-assisted services and pet–human research are built. Studies have already considered the role of attachment theory in animal–human interactions, showing that both humans and animals engage in relationships in ways that both align with known concepts of relational attachment [87,109]. Furthermore, when we consider research in animal–human bonds and the corresponding behaviors, we see evidence of various levels of the proposed physical and psychological bonding present in the Interspecies Relational Theory. Basic interactions built over time become part of the unique bonds between a human and nonhuman animal and can be developed over time to mimic human–human bonding. For example, time spent with a nonhuman animal building positive interactions results in positive associations, as seen with horses [95]. Cats show behaviors with their primary humans that mimic human attachment [87] and the dog–human bond has been well studied in both the behavioral indicators of attachment as well as in oxytocin indicators for both humans and dogs [110,111,112].

### 4.5. The Role of Choice

The literature on healthy friendships, including human–nonhuman animal relationships, continues to emphasize the importance of choice and autonomy [5,83,113]. Levels of distress decreases learning and performance in animals [114,115], whereas providing choiceallows for the ability of the other to control their environment, their place within the environment, and types and durations of interactions with humans which can decrease stress, promote natural behavior, and increase feelings of safety [113].

Following Maslow’s Hierarchy and studies on trust, autonomy is essential for individuals, both human and nonhuman, to act on their own subjective associations within the development of relationships in order to feel physically and emotionally safe and to continue to act as an independent agent in building mutual relationships. The role of agency in the five domain model of welfare has also been highlighted, emphasizing the importance of choice as part of interactions with both environments and other animals [113]. This suggests that autonomy supports a larger role of intrinsic motivation rather than coercion, luring, or behavioral shaping.

## 5. Related Theories

As the study of human–animal bonds and animal-assisted services have grown, so too have the theories around how these bonds are formed and the impact of these relationships on both human and nonhuman animals. While most of the studies focus exclusively on the impacts on the human, researchers have developed theories to support the subjective feelings of human participants and the potential emotional bonds experienced by nonhuman animals.

### 5.1. Attachment Theory

Attachment theory, originally developed by Bowlby (1969) and later expanded by Ainsworth and others [116], describes the formation of emotional bonds between individuals based on the need for safety, proximity, and emotional regulation. Central to the theory are concepts such as secure base behavior, responses of primary caregivers to the needs of those in their care, and attachment styles that shape future relational expectations. While Attachment theory has been applied to some human–nonhuman animal relationships, it traditionally reflects how current relational dynamics and emotional security reflect past experiences. While Interspecies Relational Theory takes into consideration individual experiences and the impact these may have on the development of new relationships, Interspecies Relational Theory emphasizes mutual agency and co-constructed relational development between species rather than emphasizing specific roles in the relationship. Rather than assuming hierarchical caregiving roles, Interspecies Relational Theory proposes that both human and nonhuman animals actively shape the relationship through behavior, communication, and contextual trust-building. It incorporates concepts of attachment theory, extends the logic of attachment to account for species-specific and individual variations in behaviors, and emphasizes relationship dynamics that emerge over time through shared proximity, shared language, and mutual choice.

### 5.2. Relational Ecology

Interspecies Relational Theory builds upon broader frameworks such as Putney’s (2013) relational ecology, which emphasizes the role of relationships in shaping individual wellbeing [117]. Relational ecology offers a systems-level perspective on connection and embeddedness within ecological and social networks, considering the impact of the vast connection of relationships, including those with nonhuman animals, on the individual. Interspecies Relational Theory provides a more structured, behaviorally grounded model focused specifically on the development of interspecies relationships and the interactions between two individuals that develop over time. By emphasizing agency, proximity, and mutual communication, Interspecies Relational Theory complements relational ecology by detailing how trust and social bonds emerge within the greater framework of social systems.

## 6. Conclusions

While the framework offers conceptual clarity, empirical validation across species and contexts is still needed. Additionally, species-specific social structures, lifespans, and cognition may limit the generalizability of certain stages of relationship development. Individual variation, although emphasized in the application of the framework, will mean that the usability of the concepts will vary greatly between individuals and different interspecies relationships. Variations in individual histories and experiences in relationships will mean that the stages of interspecies relationship development will play out differently for each dyad.

As concepts of one health and one welfare become more popular as a way of contextualizing our effect on others and others’ effects on us [4], we need to take a closer look at the types of relationships we have with other beings and how we can better understand and develop these relationships as mutually beneficial not just in production and exchange, but also at a social and emotional level. By deconstructing what we know about relationships between people and comparing them to what we know about relationships and friendships in animals, we can develop a sense of the components of these relationships and how they can be used in domestic settings to facilitate interactions that are seen as emotional and social growth by both humans and nonhumans. The development of an Interspecies Relational Theory provides a framework for building stronger social bonds between humans and nonhuman animals. This is especially critical in animal-assisted and veterinary fields where interspecies interactions sit at the core of the work.

## Data Availability

Not applicable.

## References

[1] Prato-Previde E., Basso Ricci E., Colombo E.S. (2022). The Complexity of the Human–Animal Bond: Empathy, Attachment and Anthropomorphism in Human–Animal Relationships and Animal Hoarding. Animals.

[2] Luke K.L., Rawluk A., McAdie T. (2022). A New Approach to Horse Welfare Based on Systems Thinking. Anim. Welf..

[3] Chandler C.K. (2018). Human-Animal Relational Theory: A Guide for Animal-Assisted Counseling. J. Creat. Ment. Health.

[4] Tarazona A.M., Ceballos M.C., Broom D.M. (2020). Human Relationships with Domestic and Other Animals: One Health, One Welfare, One Biology. Animals.

[5] Rault J.L., Waiblinger S., Boivin X., Hemsworth P. (2020). The Power of a Positive Human–Animal Relationship for Animal Welfare. Front. Vet. Sci..

[6] Kieson E., Abramson C.I. (2016). Equines as Tools vs. Partners: A Critical Look at the Uses and Beliefs Surrounding Horses in Equine Therapies and Argument for Mechanical Horses. J. Vet. Behav. Clin. Appl. Res..

[7] Hawkins E., Hawkins R., Dennis M., Williams J., Lawrie S.M. (2019). Animal-Assisted Therapy, Including Animal-Assisted Activities and Resident Animals, for Improving Quality of Life in People with Stroke. Cochrane Database Syst. Rev..

[8] Hatch A. (2007). The View from All Fours: A Look at an Animal-Assisted Activity Program from the Animals’ Perspective. Anthrozoos.

[9] Wijnen B., Martens P. (2022). Animals in Animal-Assisted Services: Are They Volunteers or Professionals?. Animals.

[10] Ng Z., Morse L., Albright J., Viera A., Souza M. (2019). Describing the Use of Animals in Animal-Assisted Intervention Research. J. Appl. Anim. Welf. Sci..

[11] Beck A.M. (2000). The use of animals to benefit humans: Animal-assisted therapy. Handbook on Animal-Assisted Therapy: Theoretical Foundations and Guidelines for Practice.

[12] Walsh F. (2009). Human-Animal Bonds I: The Relational Significance of Companion Animals. Fam. Process.

[13] Massen J.J.M. (2018). Friendships in Animals. Encyclopedia of Animal Cognition and Behavior.

[14] Wolter R., Stefanski V., Krueger K. (2018). Parameters for the Analysis of Social Bonds in Horses. Animals.

[15] Ricci-Bonot C., Kiley-Worthington M. (2017). The Roles of Individuals and Social Networking in a Small Group of Domestic Horses at Pasture. J. Anim. Health Behav. Sci..

[16] Silk J. (2016). Animal Behaviour: Friendship Enhances Trust in Chimpanzees. Curr. Biol..

[17] Koski S.E., Scott R., Kosslyn S. (2015). Reconciliation and Peace-Making: Insights from Studies on Nonhuman Animals. Emerging Trends in the Social and Behavioral Sciences.

[18] Cafazzo S., Marshall-Pescini S., Lazzaroni M., Virányi Z., Range F. (2018). The Effect of Domestication on Post-Conflict Management: Wolves Reconcile While Dogs Avoid Each Other. R. Soc. Open Sci..

[19] Brosnan S.F., Bshary R. (2010). Cooperation and Deception: From Evolution to Mechanisms. Philos. Trans. R. Soc. B Biol. Sci..

[20] Trivers R.L. (1970). The Evolution of Reciprocal Altruism. Q. Rev. Biol..

[21] Cooke S. (2019). Betraying Animals. J. Ethics.

[22] Mellor D.J., Beausoleil N.J., Littlewood K.E., McLean A.N., McGreevy P.D., Jones B., Wilkins C. (2020). The 2020 Five Domains Model: Including Human–Animal Interactions in Assessments of Animal Welfare. Animals.

[23] García Pinillos R., Appleby M.C., Manteca X., Scott-Park F., Smith C., Velarde A. (2016). One Welfare—A Platform for Improving Human and Animal Welfare. Vet. Rec..

[24] Mota-Rojas D., Monsalve S., Lezama-García K., Mora-Medina P., Domínguez-Oliva A., Ramírez-Necoechea R., Garcia R.D.C.M. (2022). Animal Abuse as an Indicator of Domestic Violence: One Health, One Welfare Approach. Animals.

[25] Griffin K.E., Arndt S.S., Vinke C.M. (2023). The Adaptation of Maslow’s Hierarchy of Needs to the Hierarchy of Dogs’ Needs Using a Consensus Building Approach. Animals.

[26] Kenrick D.T., Griskevicius V., Neuberg S.L., Schaller M. (2010). Renovating the Pyramid of Needs: Contemporary Extensions Built upon Ancient Foundations. Perspect. Psychol. Sci..

[27] Ghaleb B.D.S. (2024). Towards A Dynamic Model of Human Needs: A Critical Analysis of Maslow’s Hierarchy. Int. J. Multidiscip. Approach Res. Sci..

[28] Schilke O., Reimann M., Cook K.S. (2021). Trust in Social Relations. Annu. Rev. Sociol..

[29] Mayer R.C., Davis J.H., David Schoorman F. (1995). An Integrative Model of Organizational Trust. Acad. Manag. Rev..

[30] Hancock P.A., Kessler T.T., Kaplan A.D., Stowers K., Brill J.C., Billings D.R., Schaefer K.E., Szalma J.L. (2023). How and Why Humans Trust: A Meta-Analysis and Elaborated Model. Front. Psychol..

[31] Rault J. (2019). Be Kind to Others: Prosocial Behaviours and Their Implications for Animal Welfare. Appl. Anim. Behav. Sci..

[32] Lewicki R.J., Bunker B.B. (1996). Developing and Maintaining Trust in Work Relationships. Trust in Organizations: Frontiers of Theory and Research.

[33] McAllister D.J., Lewicki R.J., Chaturvedi S. (2006). Trust in Developing Relationships: From Theory To Measurement. Acad. Manag. Proc..

[34] Rotenberg K.J. (2010). Interpersonal Trust During Childhood and Adolescence.

[35] Forss S., Ciria A., Clark F., Galusca C., Harrison D., Lee S. (2024). A Transdisciplinary View on Curiosity beyond Linguistic Humans: Animals, Infants, and Artificial Intelligence. Biol. Rev..

[36] Searcy W.A. (2019). Animal Communication, Cognition, and the Evolution of Language. Anim. Behav..

[37] Brainard M.S., Fitch W.T. (2014). Editorial Overview: Communication and Language: Animal Communication and Human Language. Curr. Opin. Neurobiol..

[38] Merkies K., Franzin O. (2021). Enhanced Understanding of Horse–Human Interactions to Optimize Welfare. Animals.

[39] Mealand B., Rigg S., Shelton L., Wood L. (2025). What Matters Most: The Horse’s Experience of the Experience. Anim. Behav. Welf. Cases.

[40] Stankowich T., Blumstein D.T. (2005). Fear in Animals: A Meta-Analysis and Review of Risk Assessment. Proc. R. Soc. B Biol. Sci..

[41] Weiss A., Michels C., Burgmer P., Mussweiler T., Ockenfels A., Hofmann W. (2021). Trust in Everyday Life. J. Pers. Soc. Psychol..

[42] Wess L., Böhm A., Schützinger M., Riemer S., Yee J.R., Affenzeller N., Arhant C. (2022). Effect of Cooperative Care Training on Physiological Parameters and Compliance in Dogs Undergoing a Veterinary Examination—A Pilot Study. Appl. Anim. Behav. Sci..

[43] Carroll S.L., Sykes B.W., Mills P.C. (2022). Moving toward Fear-Free Husbandry and Veterinary Care for Horses. Animals.

[44] Demaline B. (2018). Fear in the Veterinary Clinic: History and Development of the Fear Free^SM^ Initiative. Conspec. Boreal..

[45] Rempel J.K., Holmes J.G., Zanna M.P. (1985). Trust in Close Relationships. J. Pers. Soc. Psychol..

[46] Panksepp J. (2005). Affective Consciousness: Core Emotional Feelings in Animals and Humans. Conscious. Cogn..

[47] Pelgrim M.H., Espinosa J., Tecwyn E.C., Marton S.M.K., Johnston A., Buchsbaum D. (2021). What’s the Point? Domestic Dogs’ Sensitivity to the Accuracy of Human Informants. Anim. Cogn..

[48] Bosmans G., Waters T.E.A., Finet C., de Winter S., Hermans D. (2019). Trust Development as an Expectancy-Learning Process: Testing Contingency Effects. PLoS ONE.

[49] Eubanks C.F. (2022). Rupture Repair. Cogn. Behav. Pract..

[50] Aragunde-Kohl U., Gómez-Galán J., Lázaro-Pérez C., Martínez-López J.Á. (2020). Interaction and Emotional Connection with Pets: A Descriptive Analysis from Puerto Rico. Animals.

[51] Shoib S., Hussaini S.S., Chandradasa M., Saeed F., Khan T., Swed S., Lengvenyte A. (2022). Role of Pets and Animal Assisted Therapy in Suicide Prevention. Ann. Med. Surg..

[52] Amiot C.E., Bastian B. (2023). What Is Beneficial in Our Relationships with Pets? Exploring the Psychological Factors Involved in Human–Pet Relations and Their Associations with Human Wellbeing. Anthrozoos.

[53] Ujita A., Seekford Z., Kott M., Goncherenko G., Dias N.W., Feuerbacher E., Bergamasco L., Jacobs L., Eversole D.E., Negrão J.A. (2021). Habituation Protocols Improve Behavioral and Physiological Responses of Beef Cattle Exposed to Students in an Animal Handling Class. Animals.

[54] Lundberg P., Hartmann E., Roth L.S.V. (2020). Does Training Style Affect the Human-Horse Relationship? Asking the Horse in a Separation–Reunion Experiment with the Owner and a Stranger. Appl. Anim. Behav. Sci..

[55] Cantor M., Farine D.R., Daura-Jorge F.G. (2023). Foraging Synchrony Drives Resilience in Human-Dolphin Mutualism. Proc. Natl. Acad. Sci. USA.

[56] Spottiswoode C.N., Begg K.S., Begg C.M. (2016). Reciprocal Signaling in Honeyguide-Human Mutualism. Science.

[57] McLean A.N., Christensen J.W. (2017). The Application of Learning Theory in Horse Training. Appl. Anim. Behav. Sci..

[58] Doherty O., McGreevy P.D., Pearson G. (2017). The Importance of Learning Theory and Equitation Science to the Veterinarian. Appl. Anim. Behav. Sci..

[59] Starling M.J., Branson N., Cody D., McGreevy P.D. (2013). Conceptualising the Impact of Arousal and Affective State on Training Outcomes of Operant Conditioning. Animals.

[60] Ninomiya S., Mitsumasu T., Aoyama M., Kusunose R. (2007). A Note on the Effect of a Palatable Food Reward on Operant Conditioning in Horses. Appl. Anim. Behav. Sci..

[61] Beery A.K., Kaufer D. (2015). Stress, Social Behavior, and Resilience: Insights from Rodents. Neurobiol. Stress..

[62] Balliet D., Van Lange P.A.M. (2013). Trust, Conflict, and Cooperation: A Meta-Analysis. Psychol. Bull..

[63] Steinbeis N., Engert V., Linz R., Singer T. (2015). The Effects of Stress and Affiliation on Social Decision-Making: Investigating the Tend-and-Befriend Pattern. Psychoneuroendocrinology.

[64] Cardoso C., Ellenbogen M.A., Serravalle L., Linnen A.M. (2013). Stress-Induced Negative Mood Moderates the Relation between Oxytocin Administration and Trust: Evidence for the Tend-and-Befriend Response to Stress?. Psychoneuroendocrinology.

[65] Taylor S., Master S. (2011). Social Responses to Stress: The Tend and Befriend Model. Handb. Stress Sci. Biol. Psychol. Health.

[66] Taylor S.E. (2006). Tend and Befriend Biobehavioral Bases of Affiliation Under Stress. Curr. Dir. Psychol. Sci..

[67] Kieson E., Goma A.A., Radi M. (2023). Tend and Befriend in Horses: Partner Preferences, Lateralization, and Contextualization of Allogrooming in Two Socially Stable Herds of Quarter Horse Mares. Animals.

[68] Komiya A., Ozono H., Watabe M., Miyamoto Y., Ohtsubo Y., Oishi S. (2020). Socio-Ecological Hypothesis of Reconciliation: Cultural, Individual, and Situational Variations in Willingness to Accept Apology or Compensation. Front. Psychol..

[69] Palagi E., Norscia I. (2015). The Season for Peace: Reconciliation in a Despotic Species (*Lemur catta*). PLoS ONE.

[70] De Waal F.B.M. (2000). Primates-A Natural Heritage of Conflict Resolution. Science.

[71] Takaoka A., Maeda T., Hori Y., Fujita K. (2015). Do Dogs Follow Behavioral Cues from an Unreliable Human?. Anim. Cogn..

[72] De Castro A.C.V., Fuchs D., Morello G.M., Pastur S., De Sousa L., Olsson I.A.S. (2020). Does Training Method Matter? Evidence for the Negative Impact of Aversive-Based Methods on Companion Dog Welfare. PLoS ONE.

[73] Cavalli C., Dzik V., Carballo F., Bentosela M. (2016). Post-Conflict Affiliative Behaviors Towards Humans in Domestic Dogs (Canis Familiaris). Int. J. Comp. Psychol..

[74] Gingins S., Werminghausen J., Johnstone R.A., Grutter A.S., Bshary R. (2013). Power and Temptation Cause Shifts between Exploitation and Cooperation in a Cleaner Wrasse Mutualism. Proc. R. Soc. B Biol. Sci..

[75] Mills S.C., Côté I.M. (2010). Crime and Punishment in a Roaming Cleanerfish. Proceedings of the Royal Society B: Biological Sciences.

[76] Bshary R., Grutter A.S. (2005). Punishment and Partner Switching Cause Cooperative Behaviour in a Cleaning Mutualism. Biol. Lett..

[77] Lansade L., Bonneau C., Parias C., Biau S. (2019). Horse’s Emotional State and Rider Safety during Grooming Practices, a Field Study. Appl. Anim. Behav. Sci..

[78] Goodwin D., McGreevy P., Waran N., McLean A. (2009). How Equitation Science Can Elucidate and Refine Horsemanship Techniques. Vet. J..

[79] Burgon H.L. (2014). Horses, Mindfulness and the Natural Environment: Observations from a Qualitative Study with at-Risk Young People Participating in Therapeutic Horsemanship. Int. J. Psychosoc. Rehabil..

[80] Çetinkaya E., Kemer G., Bulgan G., Tezer E. (2008). The Validity and Reliability Studies of Dyadic Trust Scale. Turk. Psychol. Couns. Guid. J..

[81] Parkinson C., Kleinbaum A.M., Wheatley T. (2018). Similar Neural Responses Predict Friendship. Nat. Commun..

[82] Rodrigues M.A., Yoon S.O., Clancy K.B.H., Stine-Morrow E.A.L. (2021). What Are Friends for? The Impact of Friendship on Communicative Efficiency and Cortisol Response during Collaborative Problem Solving among Younger and Older Women. J. Women Aging.

[83] Pezirkianidis C., Galanaki E., Raftopoulou G., Moraitou D., Stalikas A. (2023). Adult Friendship and Wellbeing: A Systematic Review with Practical Implications. Front. Psychol..

[84] Bhattacharjee D., Flay K.J., McElligott A.G. (2024). Personality Homophily Drives Female Friendships in a Feral Ungulate. iScience.

[85] Heitor F., do Mar Oom M., Vicente L. (2006). Social Relationships in a Herd of Sorraia Horses. Part II. Factors Affecting Affiliative Relationships and Sexual Behaviours. Behav. Process..

[86] Burkhard M.E., Range F., Ward S.J., Robinson L.M. (2023). Bonded by Nature: Humans Form Equally Strong and Reciprocated Bonds with Similar Raised Dogs and Wolves. Front. Psychol..

[87] Vitale K.R., Behnke A.C., Udell M.A.R. (2019). Attachment Bonds between Domestic Cats and Humans. Curr. Biol..

[88] Fine A., Kogan L., Blazina C. (2019). The Human-Animal Bond Over the Lifespan: A Primer for Mental Health Professionals. Clinician’s Guide to Treating Companion Animal Issues: Addressing Human-Animal Interaction.

[89] Melson G.F. (2003). Child Development and the Human-Companion Animal Bond. Am. Behav. Sci..

[90] Evans Robino A. (2019). The Human-Animal Bond and Attachment in Animal-Assisted Interventions in Counseling. Ph.D. Thesis.

[91] Hosey G., Melfi V. (2014). Human-Animal Interactions, Relationships and Bonds: A Review and Analysis of the Literature. Int. J. Comp. Psychol..

[92] Kerepesi A., Doka A., Miklosi A. (2015). Dogs and Their Human Companions: The Effect of Familiarity on Dog-Human Interactions. Behav. Process..

[93] Rehn T., Keeling L.J. (2016). Measuring Dog-Owner Relationships: Crossing Boundaries between Animal Behaviour and Human Psychology. Appl. Anim. Behav. Sci..

[94] Beck A.M., Katcher A.H. (2003). Future Directions in Human-Animal Bond Research. Am. Behav. Sci..

[95] Sankey C., Richard-Yris M.-A., Leroy H., Henry S., Hausberger M. (2010). Positive Interactions Lead to Lasting Positive Memories in Horses, Equus Caballus. Anim. Behav..

[96] Jardat P., Lansade L. (2022). Cognition and the Human–Animal Relationship: A Review of the Sociocognitive Skills of Domestic Mammals toward Humans. Anim. Cogn..

[97] Carter C.S. (2017). The Oxytocin-Vasopressin Pathway in the Context of Love and Fear. Front. Endocrinol..

[98] Kumsta R., Heinrichs M. (2013). Oxytocin, Stress and Social Behavior: Neurogenetics of the Human Oxytocin System. Curr. Opin. Neurobiol..

[99] Froemke R.C., Young L.J. (2021). Oxytocin, Neural Plasticity, and Social Behavior. Annu. Rev. Neurosci..

[100] Heinrichs M., von Dawans B., Domes G. (2009). Oxytocin, Vasopressin, and Human Social Behavior. Front. Neuroendocrinol..

[101] Donaldson Z.R., Young L.J. (2008). Oxytocin, Vasopressin, and the Neurogenetics of Sociality. Science.

[102] Byrd-Craven J., Granger D.A., Auer B.J. (2016). Stress Reactivity to Co-Rumination in Young Women’s Friendships: Cortisol, Alpha-Amylase, and Negative Affect Focus. J. Soc. Pers. Relat..

[103] Insel T.R., Winslow J.T., Wang Z., Young L.J., Zingg H.H., Bourque C.W., Bichet D.G. (1998). Oxytocin, Vasopressin, and the Neuroendocrine Bass of Pair Bond Formation. Vasopressin and Oxytocin: Molecular, Cellular, and Clinical Advances.

[104] Weinstein T.A.R., Bales K.L., Maninger N., Hostetler C.M., Capitanio J.P. (2014). Early Involvement in Friendships Predicts Later Plasma Concentrations of Oxytocin and Vasopressin in Juvenile Rhesus Macaques (*Macaca mulatta*). Front. Behav. Neurosci..

[105] Burkhart J.C., Gupta S., Borrego N., Heilbronner S.R., Packer C. (2022). Oxytocin Promotes Social Proximity and Decreases Vigilance in Groups of African Lions. iScience.

[106] Nagasawa M., Mitsui S., En S., Ohtani N., Ohta M., Sakuma Y., Onaka T., Mogi K., Kikusui T. (2015). Oxytocin-Gaze Positive Loop and the Coevolution of Human-Dog Bonds. Science.

[107] Höglin A., Van Poucke E., Katajamaa R., Jensen P., Theodorsson E., Roth L.S.V. (2021). Long-Term Stress in Dogs Is Related to the Human–Dog Relationship and Personality Traits. Sci. Rep..

[108] McGreevy P.D. (2007). The Advent of Equitation Science. Vet. J..

[109] Bachi K. (2013). Application of Attachment Theory to Equine-Facilitated Psychotherapy. J. Contemp. Psychother..

[110] Kroll J. (2020). The Horse-Human Bond as Catalyst for Healing from Sexual or Domestic Abuse: Metaphors in Gillian Mears’ Foal’s Bread. Int. J. Pract. Theory Creat. Writ..

[111] Oliva J.L., Rault J.-L., Appleton B., Lill A. (2016). Oxytocin Blocks Pet Dog (Canis Familiaris) Object Choice Task Performance Being Predicted by Owner-Perceived Intelligence and Owner Attachment. Pet. Behav. Sci..

[112] Payne E., DeAraugo J., Bennett P., McGreevy P. (2016). Exploring the Existence and Potential Underpinnings of Dog-Human and Horse-Human Attachment Bonds. Behav. Process..

[113] Littlewood K.E., Heslop M.V., Cobb M.L. (2023). The Agency Domain and Behavioral Interactions: Assessing Positive Animal Welfare Using the Five Domains Model. Front. Vet. Sci..

[114] Olczak K., Nowicki J., Klocek C. (2016). Motivation, Stress and Learning—Critical Characteristics That Influence the Horses’ Value and Training Method—A Review. Ann. Anim. Sci..

[115] Brajon S., Laforest J.-P., Schmitt O., Devillers N. (2016). A Preliminary Study of the Effects of Individual Response to Challenge Tests and Stress Induced by Humans on Learning Performance of Weaned Piglets (*Sus scrofa*). Behav. Process..

[116] Bretherton I. (1992). The Origins of Attachment Theory: John Bowlby and Mary Ainsworth. Dev. Psychol..

[117] Putney J.M. (2013). Relational Ecology: A Theoretical Framework for Understanding the Human-Animal Bond. J. Sociol. Soc. Welf..

